# Multiple Coronary Artery Microfistulas Associated with Apical Hypertrophic Cardiomyopathy: Left and Right Coronary Artery to the Left Ventricle

**DOI:** 10.1155/2015/819839

**Published:** 2015-11-11

**Authors:** Jeong-Woo Choi, Kyehwan Kim, Min Gyu Kang, Jin-Sin Koh, Jeong Rang Park, Jin-Yong Hwang

**Affiliations:** Division of Cardiology, Department of Internal Medicine, Gyeongsang National University Hospital, Jinju 52727, Republic of Korea

## Abstract

A 76-year-old woman underwent coronary angiography for chest pain. On the coronary angiogram, no significant coronary artery atherosclerotic stenosis was observed. Multiple coronary artery microfistulas, draining from the left anterior descending artery to the left ventricle and from the posterior descending artery of the right coronary artery to the left ventricle, were observed. Apical wall thickening and fistula flow from the left anterior descending artery were demonstrated by using transthoracic echocardiography. We describe a rare case of multiple coronary artery microfistulas from the left and right coronary artery to the left ventricle combined with apical hypertrophic cardiomyopathy.

## 1. Introduction

Coronary artery fistula (CAF) has been known as a rare entity of cardiac anomaly. The incidence of CAF has been reported to be about 0.1–0.2% [1]. A recent retrospective study reported a prevalence of up to 2% in patients who had undergone diagnostic angiography [2, 3]. However, CAFs from both coronary arteries that drain into the left ventricle (LV) are rare. Moreover, apical hypertrophic cardiomyopathy (AHCM) coexisting with CAF has been rarely reported [4–8]. We report a rare case of CAF associated with AHCM that presented with chest pain.

## 2. Case

**Figure 1:**
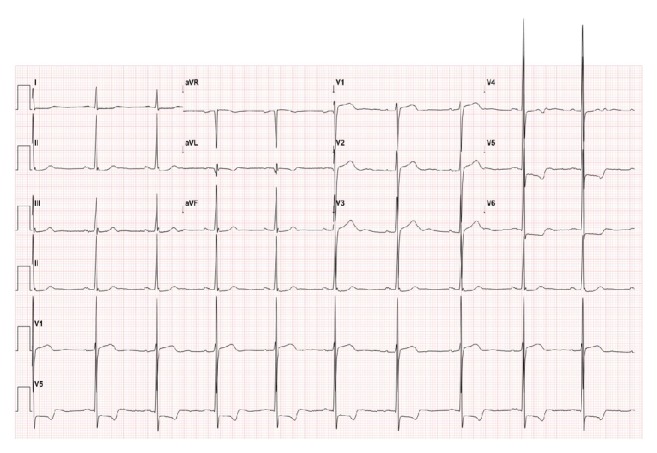
Electrocardiography. Baseline electrocardiogram showing left ventricular hypertrophy and ST-T changes in precordial leads 4–6.

A 76-year-old woman without histories of cardiovascular risks such as ischemic heart disease and heart failure and use of medications was referred to our hospital because of chest pain. Her chest pain was characterized by a squeezing sensation that was unrelated to exercise and lasted for less than half an hour. She denied any family history of heart disease. Her initial blood pressure was 110/60 mm Hg; heart rate was 68 beats per minute and regular. Auscultation revealed no evidence of cardiac murmur or crackle. On the chest radiograph, no remarkable findings such as cardiomegaly and pulmonary congestion were found. Electrocardiography revealed an ST depression in leads V5 and V6 and left ventricular hypertrophy (Figure 1). Biochemical laboratory test results, including for cardiac enzyme and B-type natriuretic peptide, were within the reference range.

**Figure 2:**
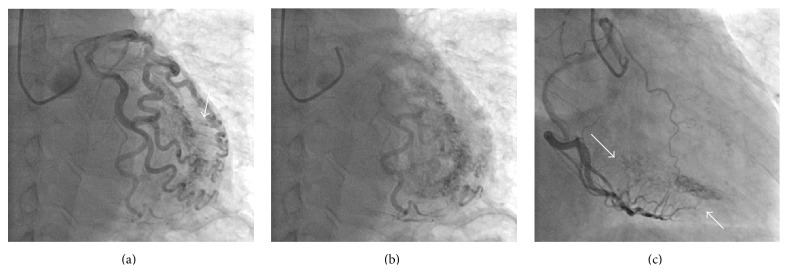
Coronary angiography. (a) A plexiform small coronary artery fistula (arrow) from the left coronary artery to the left ventricle. The left coronary artery was tortuous but showed no significant stenosis. (b) After left coronary angiography, contrast medium was drained into the left ventricle, thereby showing the endocardial border of the left ventricle. (c) Angiogram of the right coronary artery showing multiple microfistulas.

In order to exclude ischemic heart disease, invasive coronary angiography was performed. No significant luminal stenosis was observed in both the left and right coronary arteries. After contrast medium injection through the orifice of the left coronary artery, remarkable tortuosity of the left coronary artery and a plexiform network of vessels in the LV wall were observed (Figure 2(a)). The LV cavity directly communicated with the diagonal branch of the left anterior descending (LAD) artery through multiple microfistulas. After diastole, the LV was filled with contrast, enough for yielding findings on ventriculography (Figure 2(b)). The right coronary angiogram showed that the posterior descending artery of the right coronary artery was also directly drained into the LV (Figure 2(c)).

**Figure 3:**
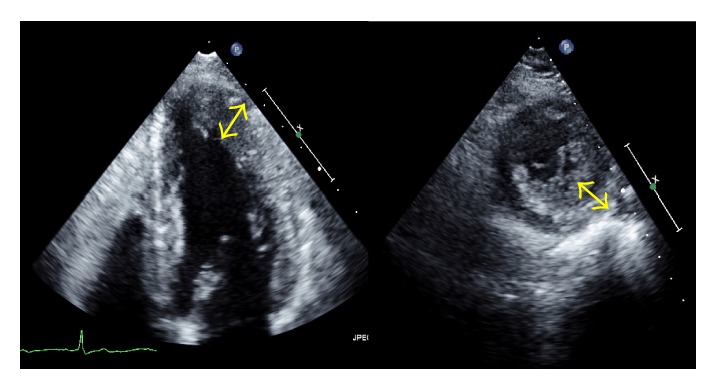
Two-dimensional echocardiography. Hypertrophy of the apical lateral wall was observed in the apical four chamber view (left) and short axis view (right).

**Figure 4:**
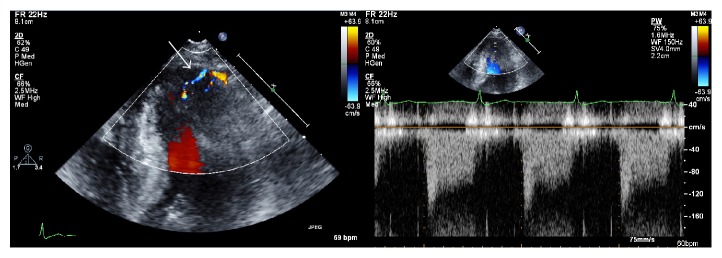
Doppler echocardiography. Color signal visible through the multiple coronary artery fistulas, which directly communicated with the left ventricle, as seen in the apical four-chamber view (left). Pulsed wave Doppler image confirming the diastolic flow of the coronary artery fistulas (right).

On two-dimensional echocardiography, the ratio of the apical lateral wall thickness (12 mm) to the left ventricular lateral free wall thickness (7 mm) was greater than 1.5 times (Figure 3). Apical obliteration, aneurysmal change, or intraventricular pressure gradient was not found. Doppler echocardiography demonstrated that color signal of perpendicular to epicardium through multiple CAFs, which directly communicated with the LV at diastole (Figure 4). Pulsed wave Doppler imaging revealed the diastolic flow of the CAFs, identified by placing a sample volume in the suspicious multiple fistulas of the myocardium (Figure 4). The size of the LV was within the normal range, as were the end-diastolic dimension of 52 mm and end-diastolic volume of 57 mL. The ratio of the mitral inflow *E* velocity to the annular *e*′ velocity was 6.1. No evidence of pulmonary hypertension was found.

She received calcium channel and beta-blockers. She had no chest pain during follow-up at 18 months.

## 3. Discussion

CAF is an uncommon congenital anomaly that arises from one or more coronary arteries and enters into the cardiac chamber. The incidence of CAF is low at 0.08% to 2% [1, 2], even though it varied according to time reported and ethnicity. Recently, owing to the development of cardiac imaging tools and easier access for invasive coronary angiography, the incidence of CAF has increased. However, the incidence of multiple CAFs originating from both coronary arteries is only 3–5% of all CAF cases [9], and cases with coexisting AHCM are rare. CAF termination most frequently occurs in the right ventricle, right atrium, and pulmonary artery, being low-pressure chambers [1]. The incidence of CAF draining into the LV is only 3–17% [9]. Wearn et al. categorized CAFs into three anatomical types as follows [1, 10]: Type I, arterioluminal: the fistula drains directly from the coronary arteries to the lumen of a heart chamber; Type II, arteriosinusoidal: the fistula drains from the coronary arteries via the myocardial sinusoids into the lumen of a ventricle; the communication is through the myocardial sinusoidal network; Type III, arteriocapillary: the fistula drains into the capillaries and then through the Thebesian system into a cardiac chamber. The microfistulas in our patient may be classified as the arteriosinusoidal type. The pathogenesis of CAFs is congenital in most cases. CAFs develop because of an “embryologic arrest” of normal closure of the intertrabecular spaces that connect the coronary arteries, veins, and cardiac trabeculae [1]. However, the relationship between AHCM and CAFs has not been clarified. The apical hypertrophy could be the result of chronic LV volume overload through CAFs or could be the cause of multiple CAFs, possibly due to the disarray of myocardial cells. One retrospective study [2] reported 20 patients with micro-CAFs, of whom 18 (90%) had concentric LV hypertrophy, not apical hypertrophy. They suggested that volume overload over a long period can induce reactive myocardial hypertrophy. However, in our case, the LV volume was within the normal range and localized asymmetric hypertrophy in the apical lateral wall was observed, although the shunt was relatively large. Therefore, we thought that AHCM and CAFs may be related with congenital, myocardial disarray. Magnetic resonance imaging or cardiac biopsy could help to determine the pathophysiological mechanism underlying the interrelationship between CAFs and cardiomyopathies. However, we did not recommend cardiac magnetic resonance imaging for the patient because it has no additional affect to clinical management.

More than half of patients with CAFs have been reported to be possibly completely asymptomatic [1]. The reported clinical presentations are angina, atypical chest pain, syncope, dyspnea, palpitation, congestive heart failure, arrhythmia, and rarely sudden cardiac death due to rupture of an aneurysm [1]. Anginal symptoms could result from the coronary steal phenomenon. Especially in patients with CAFs with hypertrophy, aggravated imbalance of oxygen demand/supply in the myocardium can lead to chest pain and myocardial ischemia. Clinical suspicion and diagnosis of CAFs are difficult. Presence of myocardial ischemia may be confirmed by using a treadmill test and myocardial perfusion single-photon emission computed tomography [9, 11, 12], but these methods have limited diagnostic capabilities. Still, in most cases, CAFs are identified during routine coronary angiography and emergent catheterizations [12]. With the development of the resolution of coronary computed tomography, noninvasive methods for diagnosis of CAFs including microfistulas have become available [13, 14]. Therefore, we think that the role of echocardiography emerged to be more important in determining whether invasive coronary angiography is required. Dresios et al. [4] reinforced the role of Doppler echocardiography in multimodalities for cardiac imaging.

No guideline has been established for the management of patients with micro-CAFs. Conventional medical management is essential. Beta-blockers, calcium channel blockers, or nitrate is usually recommended for ischemia. Our patient was treated medically and remained asymptomatic.

In conclusion, we report a rare case of multiple microfistulas of both coronary arteries that drained into the LV associated with AHCM. In addition, close inspection of color and flow patterns on Doppler echocardiography is important in screening for the cause of chest pain in AHCM.
